# Diagnosis of Oral Squamous Cell Carcinoma Using Deep Neural Networks and Binary Particle Swarm Optimization on Histopathological Images: An AIoMT Approach

**DOI:** 10.1155/2022/6364102

**Published:** 2022-09-30

**Authors:** Mohanad A. Deif, Hani Attar, Ayman Amer, Ismail A. Elhaty, Mohammad R. Khosravi, Ahmed A. A. Solyman

**Affiliations:** ^1^Department of Bioelectronics, Modern University for Technology and Information (MTI), Cairo, Egypt; ^2^Department of Energy Engineering, Zarqa University, Zarqa, Jordan; ^3^Department of Nutrition and Dietetics, Faculty of Health Sciences, Istanbul Gelisim University, Istanbul, Turkey; ^4^Department of Computer Engineering, Persian Gulf Engineering, Bushehr, Iran; ^5^Department of Electrical and Electronics Engineering, Faculty of Engineering and Architecture, Nişantaşi University, 34398, Istanbul, Turkey

## Abstract

Overall prediction of oral cavity squamous cell carcinoma (OCSCC) remains inadequate, as more than half of patients with oral cavity cancer are detected at later stages. It is generally accepted that the differential diagnosis of OCSCC is usually difficult and requires expertise and experience. Diagnosis from biopsy tissue is a complex process, and it is slow, costly, and prone to human error. To overcome these problems, a computer-aided diagnosis (CAD) approach was proposed in this work. A dataset comprising two categories, normal epithelium of the oral cavity (NEOR) and squamous cell carcinoma of the oral cavity (OSCC), was used. Feature extraction was performed from this dataset using four deep learning (DL) models (VGG16, AlexNet, ResNet50, and Inception V3) to realize artificial intelligence of medial things (AIoMT). Binary Particle Swarm Optimization (BPSO) was used to select the best features. The effects of Reinhard stain normalization on performance were also investigated. After the best features were extracted and selected, they were classified using the XGBoost. The best classification accuracy of 96.3% was obtained when using Inception V3 with BPSO. This approach significantly contributes to improving the diagnostic efficiency of OCSCC patients using histopathological images while reducing diagnostic costs.

## 1. Introduction

Oral squamous cell carcinoma (OSCC) is a diverse collection of cancers that arise from the mucosal lining of the oral cavity [1, 2], accounting for more than 90% of all oral cancers [3]. It is a subtype of head and neck squamous cell carcinoma (HNSCC), which is the world's seventh most frequent cancer [4]. The World Health Organization estimates that 657,000 fresh cases are diagnosed annually, with over 330,000 fatalities globally. OSCC frequency rates were found to be massively greater in South Asian countries. India has the biggest number of cases (one-third of cases), whereas Pakistan has the first and second most common cancers in males and females, respectively [5]. Drinking alcohol, smoking cigarettes, poor oral hygiene, human papillomavirus (HPV) exposure, genetic background, lifestyle, ethnicity, and geographical region are all risk factors.

The detection of OSCC in early stages is essential to achieve a successful therapy, increased chances for survival, and low mortality and morbidity rates [6]. With a 50% average cure rate, the OSCC has bad prognosis [7, 8]. Microscopy-based histopathological analysis of tissue samples is considered the standard method for diagnosing OSCC [9, 10]. This diagnostic pathology methodology depends on histopathologists' interpretation, which is typically slow and error-prone, limiting its clinical utility [11]. As a result, it is critical to provide effective diagnostic tools to aid pathologists in the assessment and diagnosis of OSCC.

Lately, there have been a growing number of studies on applying artificial intelligence (AI) to improve medical diagnostics. Researchers have been able to examine AI applications in medical image analysis thanks to increasing the usage of diagnostic imaging. Deep learning (DL) [12], in particular, has shown outstanding success in solving a variety of medical image processing challenges [13], specifically in the diagnostics of pathological images [14, 15]. Computer-aided diagnosis (CAD) systems based on DL have been suggested and established on a large scale for a range of cancer sorts, such as breast cancer, prostate cancer, and lung cancer ([16, 17] and [18]). Nevertheless, the research shows that DL has been seldom used to diagnose OSCC from pathological images. To recognize keratin pearls in oral histopathology images, Dev et al. employed Convolutional Neural Network (CNN) and Random Forest. For keratin area segmentation, the CNN model achieved 98.05 percent accuracy, whereas the Random Forest model spotted keratin pearls with 96.88 percent accuracy [19]. Das et al. used DL to divide oral biopsy images into different classes according to Broder's histological grading system. CNN was also proposed, which had a 97.5 percent accuracy rate (*N*) [20].

Folmsbee et al. used CNN to classify oral cancer tissue into seven types using Active Learning (AL) and Random Learning (RL) (stroma, tumor, lymphocytes, mucosa, keratin pearls, adipose, and blood). The AL's accuracy was determined to be 3.26 percent higher than that of the RL [21]. Furthermore, Martino et al. applied multiple DL architectures, such as U-Net, SegNet, U-Net with VGG16 encoder, and U-Net with ResNet50 encoder, to segment oral lesion whole slide images (WSI) into three groups (carcinoma, noncarcinoma, and nontissue). A deeper network, such as U-Net upgraded with ResNet50 as encoder, was shown to be more accurate than the original U-Net [22]. Amin et al. recently used VGG16, Inception V3, and Resnet50 were fine-tuned individually and then used in concatenation as a feature extractor to perform binary classification on oral pathology images [23].

The categorization of oral histopathological images into normal and OSCC classes was enhanced in this research. The increase is achieved by employing the notion of transfer learning to extract features from pretrained CNN models (VGG16, AlexNet, ResNet50, and Inception V3). Then, using the Binary Particle Swarm Optimization (BPSO) approach, features were selected. Finally, three different classifiers, XGBoost, KNN, and ANN, were used to determine the detection performance of OSCC and normal oral histopathology images.

## 2. Research Contributions

The proposed research investigates the following:

- Oral histopathological images introduced to extract the desired features to four various models of DL.
- Investigating the obtained features in “a” by applying the methods of metaheuristic feature selection.
- The metaheuristic feature selection method is explored to enhance the performance of the classifiers.
- Achieving the most recommended “hybrid” system that provides the highest accuracy in the stage of the making decision, which is obtained by comparing the highest performance of the proposed models for detecting OSCC.
- The final stage of the proposed research is the effect evaluation for the stain normalization on images classification.
- Based on “a” to “e,” AIoMT platforms development [24–26] is regarded as the main goal of the introduced research in the medical and health field.

The rest of the paper is organized as follows: The literature study in Section 1 goes into detail on previous successful approaches. A discussion of the method and approaches used in this study can be found in Section 3. A breakdown of the findings from the study is given in Section 4. Finally, the outcome of the investigated study is described in Section 5 before concluding the paper in Section 6.

## 3. Materials and Methods

### 3.1. Materials

**Figure 1:**
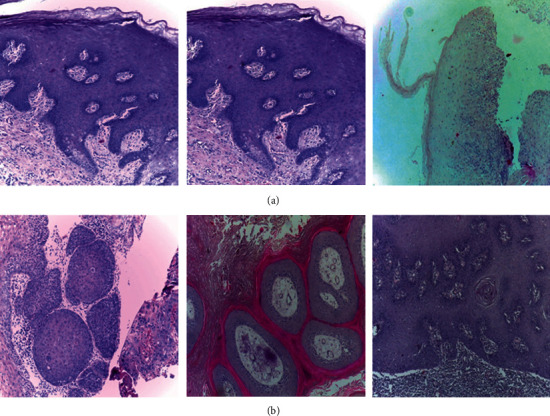
Different classes of histopathological images with (a) NEOR and (b) OSCC.

The histopathological images dataset is got from the online resource repository [27], and medical experts prepared and cataloged data from 230 individuals. These images were collected from two different locations in India: Dr. B. Borooah Cancer Research Institute and Ayursundra Healthcare Pvt. Ltd, Guwahati. Images are divided into two categories: normal epithelium of the oral cavity (NEOR) and OSCC. A Leica ICC50 HD microscope camera was used to get the images.  Table 1 shows the histopathological image details in terms of class, resolutions, and quantity.  Figure 1 shows a chosen selection of histopathological images and the related categories.

### 3.2. Proposed Model

**Figure 2:**
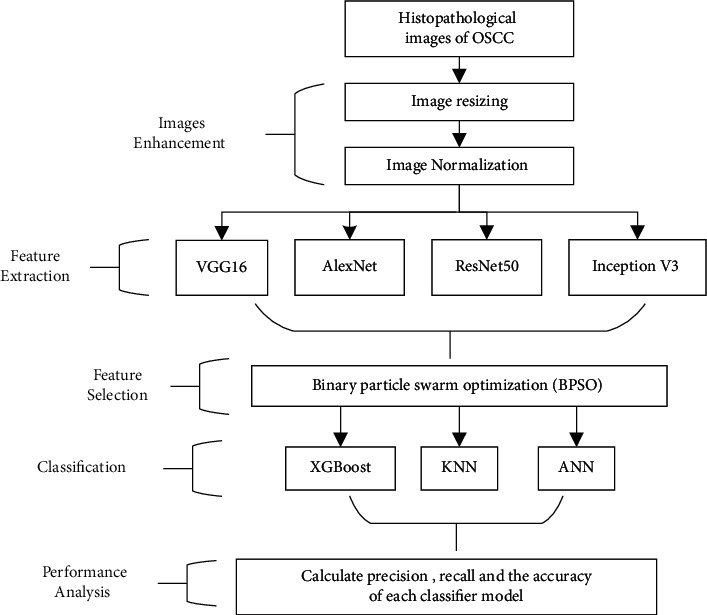
The proposed model for realizing AIoMT in medical systems.

Figure 2 depicts the proposed model. The model states that the input histopathology images were first preprocessed. In order to extract features from normalized histopathology images, four DL models were used. The transfer learning approach was used during the feature extraction procedure. To find the best probable features, Binary Particle Swarm Optimization was employed as a feature selection method. Finally, three machine learning methods were used to classify abstract features, such as DL [28].

#### 3.2.1. Image Enhancement

To develop the classification process, some preprocessing work is regarded as essential. So, the processes performed in the preprocessing step are explained in the following.

*(1) Image Resizing*. As shown in Table 2, the size of the histopathology images was adjusted depending on the size of the input of each DL architecture. Losing local features in histopathological images when resized is offset by the preservation of global features.

*(2) Image Normalization*. The analysis of the histopathology images faces may challenges, such as producing robust models to the variations resulting from different labs and imaging systems [30], caused by raw materials, the response to various colors of the scanners' slide, protocols for staining, and manufacturing techniques [31]. The histopathology dataset was stain-normalized using the Reinhard method [32, 33]. The purpose of the Reinhard stain normalization approach is to bring these stains' appearances into line [34]. This method allows a target image to be shifted in the color domain to more closely match the template image, which is applied on each histopathology image pixel as shown in equations 1 to 3 [35, 36].

Step 1: Convert both the source image *X* and target image *Y* from RGB space to *lαβ* space.
Step 2: Do the following transformation in *lαβ* space:
  (1) $$\begin{array}{c} l_{2}=\mu_{g}\left(l_{1}\right)+\left[l-\mu_{g}\left(l\right)\right]*\left[\frac{\sigma_{g}\left(l_{1}\right)}{\sigma_{g}\left(l\right)}\right]\text{,} \end{array}$$
  (2) $$\begin{array}{c} a_{2}=\mu_{g}\left(a_{1}\right)+\left[a-\mu_{g}\left(a\right)\right]*\left[\frac{\sigma_{g}\left(a_{1}\right)}{\sigma_{g}\left(a\right)}\right]\text{,} \end{array}$$
  (3) $$\begin{array}{c} b_{2}=\mu_{g}\left(b_{1}\right)+\left[b-\mu_{g}\left(b\right)\right]*\left[\frac{\sigma_{g}\left(b_{1}\right)}{\sigma_{g}\left(b\right)}\right]\text{,} \end{array}$$
where *l*_2_, *a*_2_, and*b*_2_ are intensity variables of the processed image in *lαβ* space. *l*_1_, *a*_1_, and*b*_1_ are intensity variables of target image in *lαβ* space. *l*, *a*, and*b* are intensity variables of source image. *μ*_*g*_ indicates the global mean of the image and *σ*_*g*_ represents the global standard deviation of the image.
Step 3: Convert back the processed image *Z* from *lαβ* space to RGB space.

The intensity variation of the original image is preserved using this procedure. As a result, its structure is kept, while the contrast is altered to match that of the target.

#### 3.2.2. Features Extraction Using Deep Learning Models

In this work, four DL models were used for feature extraction, namely, VGG16, AlexNet, Inception V3, and ResNet50.

**Figure 3:**
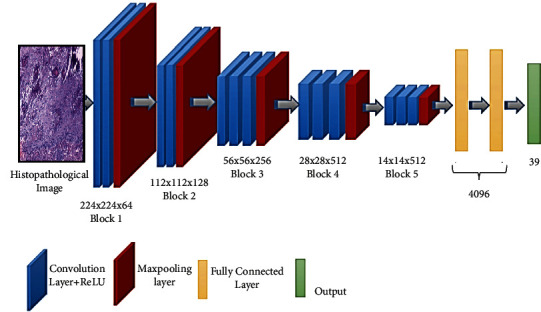
Schematic illustration of VGG16 architecture.

*(1) VGG*16. Maximum pooling and the convolution layers arrangement are followed by the VGG16 architecture [37] (Figure 3). Eventually, there are three FC layers: the first and the second are triggered by ReLU, while the third is triggered by Softmax. This design has 16 layers and 138 million parameters; the input layer can receive images with 224 × 224 pixels.

**Figure 4:**
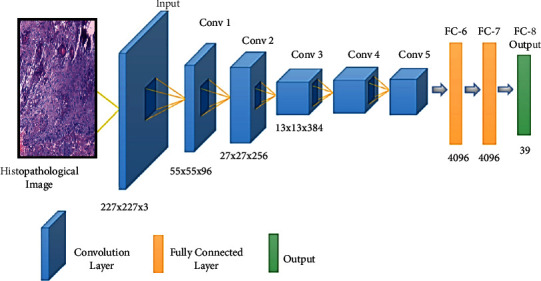
Schematic illustration of AlexNet architecture.

*(2) AlexNet*. The AlexNet [38] model contains 61 million parameters using an 8-layer CNN architecture. In the AlexNet architecture (Figure 4), there are five convolutional layers, where three of them are only used as linked layers. The fourth layer is the Softmax layer that requests a resolution of 227 × 227 pixels input image, and the last layer is the ReLU activation function, which performs the system convolutional and connected processes. Moreover, the FC-8 layer is linked to the Softmax layer via 39 neurons.

**Figure 5:**
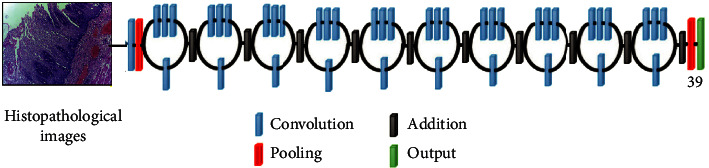
Schematic illustration of ResNet50 architecture.

*(3) ResNet*50. The ResNet50 architecture [39] addressed the issues of several nonlinear layers, not learning identity mappings, and deterioration. Simply said, ResNet50 is a network made up of residual unit stacks (Figure 5). The network is built using residual units as building components. These units were built with convolution and pooling layers. The input histopathology images have a resolution of 224 × 224 pixels, and the design includes 3 × 3 filters.

**Figure 6:**
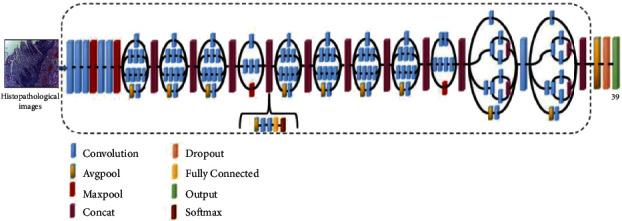
Inception V3 architecture schematic.

*(4) Inception V*3. Convolutions, average pooling, maximum pooling, dropouts, and completely connected layers are among the asymmetrical and symmetrical building elements used in the model (Figure 6). The Softmax function is found in the last layer of the Inception V3 architecture [40], which comprises 42 levels, where the resolution of the received information by the input layer in pixels is 299 × 299.

#### 3.2.3. Binary Particle Swarm Optimization for Feature Selection

**Figure 7:**
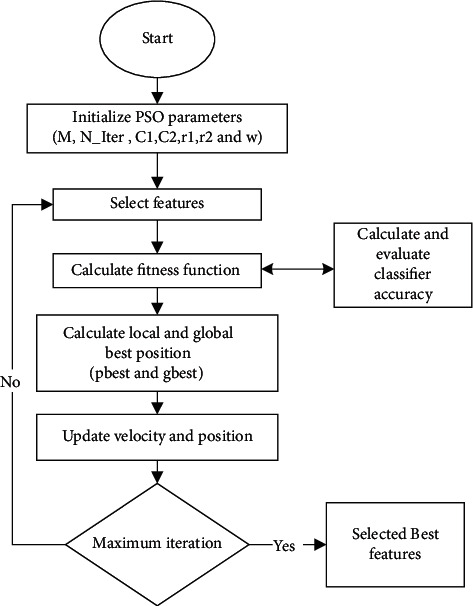
BPSO approach for feature selection.

In processing data, the selection of features is critical [41–43]. Longer training and overcompliance are challenges caused by the vast amount of data to be handled (Deif et al. [12, 44]). Unnecessary features should be removed from the data to avoid situations like this. For feature selection [7], BPSO is used, where BPSO is the binary form of PSO [45, 46]. The BPSO algorithm process was summarized in Figure 7 and described below:

Step (1): Equation (4) is used to update the particle velocity in the swarm.
  (4) $$\begin{array}{c} v_{i}^{d}\left(t+1\right)=wv_{i}^{d}\left(t\right)+c_{1}r_{1}\left(pbest_{i}^{d}\left(t\right)-x_{i}^{d}\left(t\right)\right)+c_{2}r_{2}\left(pbest^{d}\left(t\right)-x_{i}^{d}\left(t\right)\right)\text{.} \end{array}$$
Step (2): Equations (5) and (6) are used to modify the particle's location.
  (5) $$\begin{array}{c} S\left(v_{i}^{d}\left(t+1\right)\right)=\frac{1}{1+\exp\;\left(-v_{i}^{d}\left(t+1\right)\right)}\text{,} \end{array}$$
  (6) $$\begin{array}{c} x_{i}^{d}\left(t+1\right)=\left\{\begin{array}{cc} 1\text{,} & ifrand<S\left(v_{i}^{d}\left(t+1\right)\right)\text{.}\\ 0\text{,} & otherwise\text{.} \end{array}\right. \end{array}$$
Each swarm particle represents a potential solution. A 0/1 (1x feature number) matrix is one conceivable option, with each column value produced at random. Selecting the column numbers in the feature matrix with 1 yields a workable solution (a particle). The fitness value of particles in a swarm was calculated using the kNN classifier error rate. The particle with the best objective function value in the swarm is considered as *g*_best_in the first iteration.
Step (3): The particle with the highest aim function value is allocated in subsequent iterations (equation (7)).

(7) $$\begin{array}{c} Pbest\left\{\begin{array}{cc} id\left(t+1\right)=xi\left(t+1\right)\text{,} & ifF\left(x_{i}\left(t+1\right)\right)<F\left({pbest}_{i}\left(t\right)\right)\text{.}\\ {pbest}_{i}\left(t\right)\text{,} & otherwise\text{.} \end{array}\right. \end{array}$$

If *p*_best_'s aim function value is higher than *g*_best_'s, *p*_best_ is assigned to *G*_best_ (equation (8)).

(8) $$\begin{array}{c} gbest\left(t+1\right)=\left\{\begin{array}{cc} pbest_{i},\left(t+1\right)\text{,} & ifF\left({pbest}_{i}\left(t+1\right)\right)<F\left(gbest\left(t\right)\right)\text{,}\\ gbest\left(t\right)\text{,} & otherwise\text{,} \end{array}\right. \end{array}$$

where *x* is the solution, *p*_best_ denotes the personal best, *B*_best_ denotes the global best, *F*(.) denotes the fitness function, and *t* denotes the number of iterations.

This process is repeated for *T* iterations, and the algorithm returns the best global solution as output [7].

#### 3.2.4. Classification Stage

Xtreme gradient boosting (XGBoost) achieves the classification model for distinguishing between histopathological images for NEOR and OSCC. The findings of XGBoost are compared to those of classic machine learning techniques like artificial neural networks (ANN) and Random Forest (RF) [47].

#### 3.2.5. Assessment of the Suggested Methodology's Classification Performance

The suggested methodology is evaluated by calculating sensitivity, precision, and accuracy for classifiers. The following formula is used to calculate these terms:

(9) $$\begin{array}{c} Sensitivity=\frac{TP}{TP+FN}\times 100\%\text{,}\\ Precision=\frac{TP}{TP+FP}\times 100\%\text{,}\\ Accuracy=\frac{TP+TN}{TP+TN+FP+FN}\times 100\%\text{.} \end{array}$$

The number of correctly classified chest disease images in the normal and OSCC classes, respectively, is represented by TP (True Positive) and TN (True Negative). The numbers FN (False Negative) and FP (False Positive) in the normal and OSCC classes, respectively, show the number of misclassified histopathological images.

## 4. Experimental Setup and Setting

The layers of the all deep models would have been trainable true during the training phase. Softmax was used as the activation function of the final CNN layer, and MSE was used as a loss function. The early halting approach was employed, with patience set at 5 and the minimal loss change set to 0.0001. The SGD optimizer was employed in all deep models with a learning rate of 0.001. The size of the mini-batch is 32.

Histopathological images were divided into two groups in the classification stage: 80 percent for training the classifier models and 20 percent for testing, with 10-fold cross-validation. All experiments were run on Google Colab [48] with GPU support. The whole code was written in Python 3.10.1 [49] using Keras version 2.7.0 [50].

The algorithms implemented in this research are BPSO, XGBoost, ANN, and RF, taking into consideration the fact that these algorithms are varying in the hyperparameters, and the settings are shown in [51].

## 5. Results

**Figure 8:**
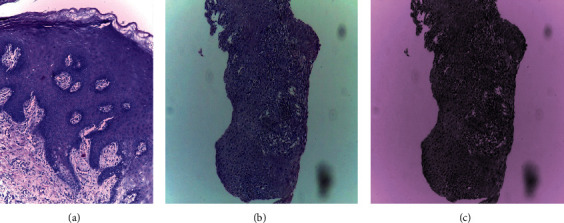
Samples of original and normalized histopathological images: (a) original image; (b) target image; and (c) Reinhard normalized.

In the first step, to better understand the impact of stain normalization approaches on classification performance, all histopathology images in the dataset were stain-normalized using the Reinhard technique. The Reinhard stain normalization approach used in this study is illustrated in Figure 8. In this diagram, (a) represents the original histopathological image used for the Reinhard technique, (b) represents the target image to be transferred (the techniques aim to normalize the colors in the target images to those of the original), and (c) represents the result of using the Reinhard technique.

**Figure 9:**
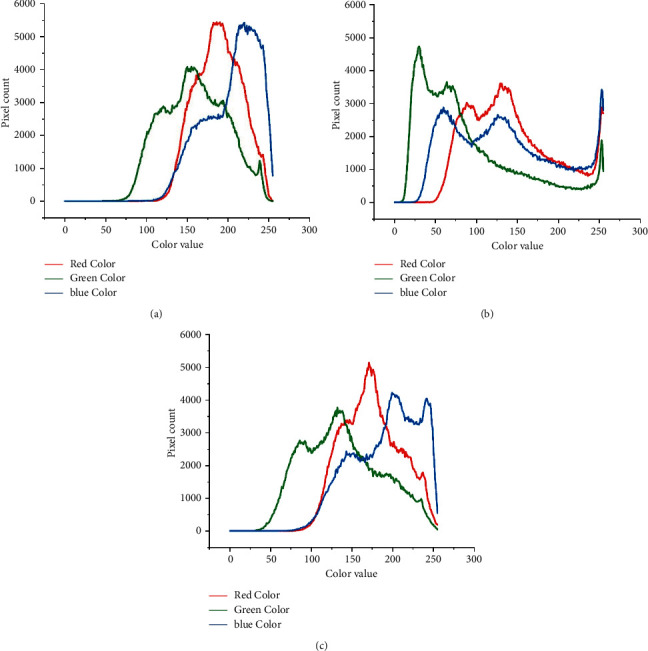
Samples of the probability density function (PDF) for (a) original histopathological image, (b) target histopathological image, and (c) Reinhard normalized.

In addition, we employed the probability density function (PDF) to investigate the effect of the Reinhard normalization method on histopathological images by comparing the postnormalization image PDF with the prenormalization image PDF.  Figure 9(a) is the original PDF. The resultant density functions of the pixels in 3-channel RGB show that the target image's distribution is substantially skewed to the left (Figure 9(b)).  Figure 9(c) shows that, after normalization, the probability distribution of the three channels resembles that of the original image.

**Figure 10:**
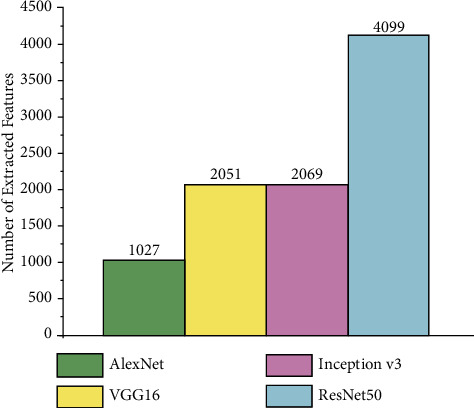
The features got in the study and all execution times.

Then, four deep models were used to extract features from the images: VGG16, AlexNet, ResNet50, and Inception V3.  Figure 10 shows the total number of features got from each deep model. It can be seen in Table 3 that the smallest number of extract features was from the AlexNet model and numerous extract features were got from the ResNet50 model. Meanwhile VGG16 and Inception V3 extracted the same number of features.

**Figure 11:**
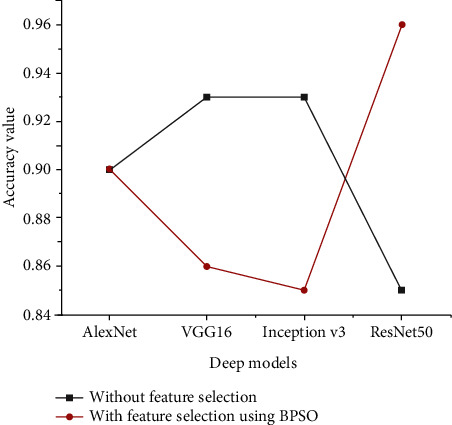
Accuracy performance for the XGBoost classifier using all extracted features and selected features.

Two experiments were carried out to see if the proposed feature selection strategy could increase the classification model's accuracy. In the first experiment, to train the classifier, all features were used without applying the feature selection technique. The second experiment was performed by employing the proposed feature selection method, that is, BPSO, to eliminate the undesired redundant and unrelated features by looking for the best features used to train the classifier. The performance values obtained for the XGBoost classifier using all extracted features and selected features are given in Figure 11.

The findings of the experiments show that when using the selected technique on features extracted using VGG16 and Inception V3, the classification performance of the XGBoost model was reduced but not when applying the selected approach on features derived using AlexNet. Meanwhile the XGBoost model delivered outstanding results when using the selected feature approach on features that were extracted using ResNet50.

**Figure 12:**
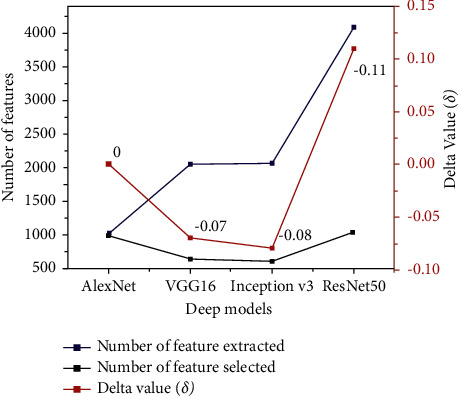
Comparison between the number of extracted features from all deep models, number of selected features, and Delta value (*δ*).

To interpret the performance of XGBoost when using the features selection technique, Figure 12 was developed to illustrate the relation between the numbers of extracted features from all deep models; the number of features was selected using BPSO and Delta value (*δ*) that indicates difference between classification accuracies for XGBoost before and after applying BPSO.

It is seen that the highest extracted number belongs to the ResNet50 model, while VGG16 and Inception V3 have about half extracted number compared to the ResNet50 model. The lowest extracted number was got from AlexNet.

It is concluded that there is a relationship between the number of features extracted and the feature selection approach, where numerous features increase the ability of BPSO to select the best features and then select from fewer features. Therefore, we can interpret that the accuracy of XGBoost increased (*δ* = 11%) when applying BPSO on features that were extracted from ResNet50 because the BPSO selects the best features from numerous features (4099) compared to features extracted from VGG16 (2051) and Inception V3 (2069), while the XGBoost accuracy has not changed (*δ* = 0).

**Figure 13:**
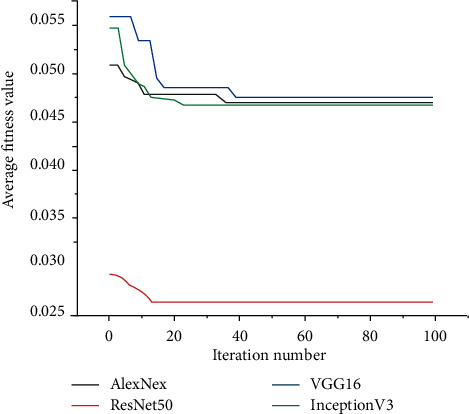
Fitness values of BPSO for different extracted features.

Figure 13 illustrates a comparison of the BPSO convergence curves algorithm at the feature selection stage. It is important to note that the fitness is the average of 20 runs. The greater the performance, the lower the values of the best fitness, worst fitness, and mean fitness. It is observed that the features obtained from VGG16 and Inception V3 models achieved the lowest fitness value for BPSO, while the best performance is given by the feature group obtained by using ResNet50.

Because the XGBoost classifier's histopathological image classification results with each features extraction method are the average accuracy got from 20 independent runs, a two-sample *t*-test with a 95 percent confidence level was used to see if the classification performance of the proposed ResNet50 with BPSO was significantly better (*p* value <0.05) than those of the other methods on histopathological images.

**Figure 14:**
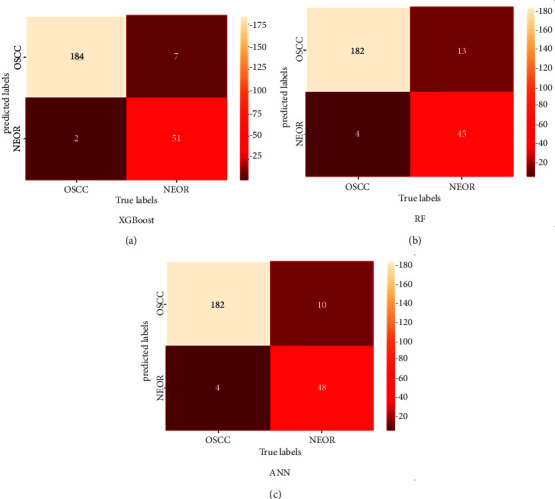
Confusion matrix of classification results for all classifiers.

To assess the XGBoost classifier's classification performance, artificial neural network (ANN) and Random Forest are two more traditional machine learning techniques that are compared to the outcomes (RF). The classifier models were trained using features taken from the ResNet50 model and then fed to the classifier by best-selected features using BPSO, based on previous results. A confusion matrix for all classifiers is shown in Figure 14.

It is clear from the confusion matrix that the highest TP results were got from the XGBoost classifier, where 184 of 186 histopathological images with OSCC were detected correctly, while two were diagnosed as NEOR. On other hand, the classifier had 7 histopathological images with NEOR. The XGBoost classifier has misclassified 7 normal histopathological images, which is the lowest number of FP compared to other classifiers.

According to the results shown in the confusion matrix, overall accuracy, sensitivity, and precision were calculated, as shown in Table 3. The XGBoost model delivered outstanding results compared to the other classifiers. It has consistently high accuracy, sensitivity, and precision (96.3%, 98.9%, and 96.3% respectively) across all models. This shows that this model was the most successful in learning and extracting essential features from the training data.

Finally, the proposed work investigates whether data normalization techniques can improve the accuracy of classification models or not, where all models were trained on nonnormalized histopathological images and the classification performance is illustrated in Table 4. It is seen that the accuracy was decreased when using histopathological images without applying the Reinhard approach. These effective results show that Reinhard stain normalization can improve the classifier performance and can produce satisfactory results.

For further evaluation of the proposed approach, it was compared with several previous research works shown in Table 5, which shows that the proposed method achieves the highest accuracy of 96.3% to classify different classes of histopathological images.

## 6. Conclusion

In this study, traditional classification algorithms are used by extracting features from the four CNN models (VGG16, AlexNet, ResNet50, and Inception V3) and selecting the best features using the BPSO algorithm. The features extracted with Inception V3 and selected with BPSO improved the classification performance and contributed positively to the results. In addition, the effects of stain normalization procedures were investigated and compared with nonnormalized histological images. The results showed that the XGBoost model performed better when the Reinhard technique was used. This breakthrough achievement has the potential to be a precious and rapid diagnostic tool that might save many people who die each year because of delayed or incorrect diagnoses.

## Figures and Tables

**Table 1:** Image categories with the related source.

Resolution	Class	Number of images
100× magnification	NEOR	89
	OSCC	439

400× magnification	NEOR	201
	OSCC	495

Total images	NEOR and OSCC	1224

**Table 2:** The input size for each DL architecture [29].

Model	Input size (pixels)
VGG16	224 × 224
AlexNet	227 × 227
ResNet50	224 × 224
Inception V3	299 × 299

**Table 3:** Performance comparison for XGBoost classifier with another state-of-the-art traditional machine learning algorithm.

Classifier name	Overall accuracy (%)	Sensitivity (%)	Precision (%)
XGBoost	96.3	98.9	96.3
RF	93.1	97.8	93.3
ANN	94.1	97.8	94.7

**Table 4:** Accuracy performance for the classifier with non-stain-normalized image and stain-normalized image

Classifier name	Without stain normalization (%)	With stain normalization (%)
XGBoost	95.1	96.3
RF	91.3	93.1
ANN	94.2	94.1

**Table 5:** Comparison of previous research works with the proposed method.

Authors	Algorithm architecture	The objective of the study	No. of images	Accuracy
Uthoff et al. [52]	CNN	Early detection of precancerous and cancerous lesions	170	86.88%
Jeyaraj et al. [53]	CNN	Develop an automated computer-aided oral cancer-detecting system	100	91.4%
Jubair et al. [54]	CNN	CNN using EfficientNet-B0 transfer model CNN for binary classication of oral lesions into benign and malignant	716	85.0%
Sunny et al. [55]	ANN	Early detection of oral potentially malignant/malignant lesion	82	86%
Rathod et al. [56]	CNN	Classify different stages of oral cancer	Not mentioned	90.68%
Alabi et al. [57]	ANNs	Predicting risk of recurrence of oral tongue squamous cell carcinoma (OTSCC)	311	81%
Alhazmi et al. [58]	ANNs	Predicting risk of developing oral cancer	73	78.95%
Chu et al. [59]	CNN	Evaluate the ability of supervised machine learning models to predict disease outcome	467	70.59%
Karadaghy et al. [60]	DT	Develop a prediction DT model using machine learning for 5-year overall survival among patients with OCSCC	33, 065	71%
Proposed method	CNN and BPSO	The features were extracted with Inception V3 and selected with BPSO to improve the classification performance of patients with OCSCC	1224	96.3%

## Data Availability

The data used to support the findings of the study can be obtained from the first author (e-mail: mohand.deif@eng.mti.edu.eg) upon request.
